# Does Cognitive Bias Modification for Appraisals Change Symptom‐Cognition Relations in PTSD? Preliminary Evidence from Network Analysis in a Randomized Controlled Trial

**DOI:** 10.1002/cpp.70308

**Published:** 2026-07-18

**Authors:** Pascal Schlechter, Thole H. Hoppen, Simon E. Blackwell, Marcella L. Woud

**Affiliations:** ^1^ Institute of Psychology University of Münster Germany; ^2^ Department of Clinical Psychology and Psychotherapy University of Göttingen Göttingen Germany; ^3^ Department of Clinical Psychology and Experimental Psychopathology University of Göttingen Göttingen Germany; ^4^ Mental Health Research and Treatment Center, Faculty of Psychology Ruhr University Bochum Bochum Germany

**Keywords:** Cognitive Bias Modification, cognitive mechanisms, dysfunctional appraisals, implicit associations, network analysis, post‐traumatic stress disorder, scenario task

## Abstract

**Background:**

Post‐traumatic stress disorder (PTSD) is maintained by dysfunctional trauma‐related appraisals. Cognitive Bias Modification for Appraisals (CBM‐APP) aims to train more functional trauma‐related appraisals and has been shown to reduce PTSD symptoms. However, little is known about how this training affects the interrelations among symptoms and cognitive appraisals.

**Methods:**

In this secondary analysis of a randomized controlled trial involving 77 adult patients diagnosed with PTSD (CBM‐APP: *n* = 37; control training: *n* = 40), we applied repeated cross‐sectional network analysis to examine changes in the structure and centrality of associations among PTSD symptom clusters (re‐experiencing, avoidance, negative cognition and mood, hyperarousal, assessed with the PTSD Checklist for DSM‐5) and trauma‐related cognitive measures, all assessed at both pre‐ and post‐training. To capture multiple levels of cognitive processing, we included responses during a scenario task (reflective, idiosyncratic, spontaneous appraisals) and the Implicit Association Test (automatic self‐associations). Four cross‐sectional Gaussian Graphical Models were estimated for the training and control group and both timepoints (pre‐/post‐training × CBM‐APP vs. control group).

**Results:**

While overall network connectivity did not differ significantly across networks, descriptive patterns indicated that *Alterations in Cognition and Mood* emerged as the most central node in both groups at post‐training assessment. Further, in the CBM‐APP group, the centrality of implicit trauma‐related associations decreased pre‐ to post‐training, suggesting potential weaker associations of automatic negative self‐associations with symptom activation.

**Conclusion:**

Given the small sample and moderate network stability, findings are preliminary but suggest that CBM‐APP may influence the relational structure of PTSD symptoms and cognitions.

## Summary

- We examined PTSD symptom‐cognition relations pre‐ and post‐training.
  • Altered Cognition and Mood became the most central node after training.
  • The centrality of implicit negative self‐associations was reduced post‐training.
  • CBM‐APP may affect symptom‐cognition interrelations.

Post‐traumatic stress disorder (PTSD) is characterized by symptoms of re‐experiencing, avoidance, negative cognition and mood and hyperarousal (American Psychiatric Association 2013). Cognitive models propose that these symptoms persist due to dysfunctional appraisals of the trauma and its aftermath (e.g., Ehlers and Clark 2000; Schnyder et al. 2015). Specifically, it is hypothesized that dysfunctional appraisals maintain distress by sustaining a sense of current threat and promoting avoidance, hypervigilance and intrusive re‐experiencing (Beierl et al. 2020; Ehlers and Clark 2000). Numerous studies highlight the role of dysfunctional appraisals in PTSD. Foa et al. 1999, for instance, developed the Posttraumatic Cognitions Inventory (PTCI) to assess trauma‐related dysfunctional appraisals about the self, world and self‐blame, finding strong links with both PTSD severity and diagnosis. Prospective research (e.g., Bryant and Guthrie 2005, 2007) has shown that a predisposition towards dysfunctional appraisals predicts later PTSD symptoms. Likewise, dysfunctional appraisals early after trauma exposure predict PTSD development months later, even after controlling for initial symptoms (e.g., Ehring et al. 2008; Kleim et al. 2007, and for an overview, see e.g., Brown et al. 2019; McNally and Woud 2019). Moreover, there is evidence that cognitive changes precede symptom reductions in PTSD in psychological treatments (e.g., Kleim et al. 2013; Schumm et al. 2022), further highlighting the centrality of cognitions in PTSD and its successful treatment.

Dysfunctional appraisals represent a central target in various evidence‐based psychotherapies for post‐traumatic stress (Hoppen et al. 2023). Extending this therapeutic focus, numerous studies have sought to manipulate or reduce such appraisals via training paradigms (e.g., computerized trainings) in experimental settings (e.g., Cheung and Bryant 2017; Hett et al. 2022; Woud et al. 2012), at risk populations (e.g., Nickerson et al. 2017; Woud, Zlomuzica, et al. 2018) and clinical populations suffering from PTSD (e.g., de Kleine et al. 2019; Woud et al. 2021). In general, results show that such trainings reduce dysfunctional appraisals and post‐traumatic stress symptoms. However, functional interrelations among symptoms and cognitive processes (i.e., how they co‐occur and co‐vary across individuals) and their change through cognitive training have not yet been investigated. To address this gap, we conducted a secondary analysis of data from a previously published randomized controlled trial (RCT) of Cognitive Bias Modification for Appraisal (CBM‐APP) in patients with PTSD (Woud et al. 2021). We aimed to examine whether and how CBM‐APP modifies interrelations among PTSD symptoms and cognitive processes, thereby elucidating putative mechanisms of action for changes in symptom patterns. Specifically, we conducted a network analysis before and after CBM‐APP (vs. a sham training control condition) to examine how this type of cognitive training influences the structure and centrality of associations among PTSD symptoms and dysfunctional appraisals. To more thoroughly understand training effects, we included multiple levels of cognitive processing, using a scenario task (Woud et al. 2019) to index spontaneous appraisals and an Implicit Association Test (IAT; Blackwell et al. 2023) assessing implicit associations via differential reaction times.

During CBM‐APP administered in clinical settings, patients are repeatedly exposed to ambiguous, trauma‐related scenarios which are then adaptively resolved by the patients in the guided computerized task, with the aim to strengthen more adaptive and more flexible appraisal tendencies. In the RCT by Woud et al. 2021, the CBM‐APP training was applied as an adjunct to treatment as usual and consisted of eight sessions over a 2‐week period. Results showed that CBM‐APP, compared to the sham training control condition, reduced both dysfunctional appraisals and PTSD symptoms. However, beyond these mean‐level changes, it is important to examine whether CBM‐APP also alters interrelations among symptoms and cognitive processes. Understanding such structural change may reveal cognitive mechanisms through which intervention effects unfold and are maintained. In this context, network analysis can be used to conceptualize symptoms and cognitive processes as interconnected nodes, with edges representing their associations (Borsboom 2017; Borsboom and Cramer 2013; McNally 2021). Network analysis enables the identification of central nodes that may activate the entire network, strong symptom intercorrelations and how these relationships reorganize post‐intervention. From this perspective, cognitive training may modify not only symptom severity but also the architecture of symptom‐cognition relations (for a review on PTSD networks, see Birkeland et al. 2020). In prior network analytic studies on PTSD, there was a large heterogeneity of central symptoms of PTSD including recurrent thoughts, negative trauma‐related emotions and intrusive memories (Birkeland et al. 2020; Schlechter et al. 2022; Sullivan et al. 2018). PTSD network studies in the context of treatment are rare, with existing studies often using treatment‐seeking samples (e.g., Duek et al. 2021; Ross et al. 2018). Studies specifically examining networks at both pre‐treatment and post‐treatment in randomized control trials are lacking, which is an important omission in current research.

Beyond self‐reported core symptoms (re‐experiencing, avoidance, negative cognition and mood, hyperarousal; American Psychiatric Association 2013), it is crucial for a network perspective to include measures on different cognitive levels. The scenario task (Woud et al. 2019) was designed to assess idiosyncratic appraisals that emerge automatically, i.e., without instructions to reflect on them, indexing spontaneous evaluative tendencies linked to real‐world trauma responses. The IAT by Blackwell et al. (2023) was designed to capture automatically activated self‐associations, such as implicit links between the self and being weak or vulnerable. Both measures are associated with self‐reported post‐traumatic stress symptoms (Blackwell et al. 2023; Woud et al. 2019). Integrating these measures with core PTSD symptom clusters allows a nuanced examination of both reflective and automatic cognitive mechanisms. This approach aligns with the dual‐process model of PTSD (Brewin et al. 1996), distinguishing fast, associative processes from slower, reflective, propositional processes. In PTSD, automatic negative self‐associations are characterized by relatively fast, associative activation, whereas reflective appraisals involve more elaborative and controlled meaning‐making about the trauma and its consequences. CBM‐APP may influence both systems: Repeated exposure to adaptive scenario resolutions may weaken dysfunctional automatic associations while reinforcing adaptive propositional appraisals. CBM‐APP targets dysfunctional trauma‐related appraisals across three domains: appraisals about the self, the world, and self‐blame. These appraisal types primarily reflect reflective, propositional meaning structures that can be deliberately accessed and modified through guided reinterpretation of ambiguous trauma‐related scenarios. Although these appraisal domains are conceptually related, their relations with implicit measures may vary. Changes in implicit self‐associations are theoretically most likely to be linked to self‐referential appraisals (Greenwald et al. 2009), whereas effects of world and self‐blame appraisals are expected to be more indirect, potentially operating via broader changes in trauma‐related meaning structures and their associations with automatic associative processes. Changes in functional connectivity between symptom clusters and cognition nodes may thus reflect increased integration between implicit and explicit systems, supporting more coherent and flexible trauma‐related representations. Thus, examining symptom‐cognition networks before and after training can reveal how CBM‐APP affects PTSD‐related processes. For instance, reducing the centrality of dysfunctional appraisals could weaken their influence on multiple other symptom clusters, by decreasing the overall connectivity of distress‐maintaining pathways. At the same time, strengthening adaptive appraisals might increase integration between reflective and automatic processes, creating new, more coherent pathways that support symptom regulation. Such structural changes could also manifest as a reduction of associations among other clusters of symptoms (e.g., re‐experiencing and hyperarousal).

In the present study, we aimed to examine whether CBM‐APP alters the network structure of PTSD symptoms and trauma‐related cognitive processes, including reflective, propositional appraisals of trauma‐relevant experiences (assessed via the scenario‐based task) and automatically activated self–trauma associations (assessed via the Implicit Association Test). By conducting a secondary network analysis to data from a RCT that has been shown to effectively reduce dysfunctional appraisals and PTSD symptoms (Woud et al. 2021), we investigated whether CBM‐APP changes the connectivity and centrality of symptom‐cognition associations. To this end, we estimated four networks (pre−/post‐training × CBM‐APP vs. sham training control group). We included three complementary modalities: (1) self‐reported PTSD symptom cluster, (2) reflective dysfunctional appraisals via a scenario‐based task (Woud et al. 2019), and (3) automatic trauma‐related associations (i.e., automatically activated self‐associations) via the IAT (Blackwell et al. 2023). These measures capture distinct but related components of post‐traumatic cognition, from deliberately endorsed cognitions to spontaneous and automatic evaluative processes. Accordingly, this study contributes to the literature by integrating multiple cognitive processing levels within a network framework, to advance understanding of how cognitive training reshapes the interrelations of PTSD symptoms and appraisals in a randomized controlled trial. Given the exploratory character of the analyses, we did not formulate a priori hypotheses.

## 1 Methods

### 1.1 Transparency and Openness

Our secondary analyses were not preregistered. However, the original trial was preregistered (clinicaltrials.gov identifier NCT02687555) and a study protocol was published (Woud, Blackwell, et al.^2018^). The R code used to reproduce the present analyses is available on the OSF, along with the dataset and codebooks (https://osf.io/zdy8b/overview). All information necessary to enable independent replication of our results is provided, as recommended (Burger et al. 2022). As this study represents a secondary analysis, we had prior access to the data, and sample sizes were determined by data availability.

### 1.2 Study Design

The current study is a secondary data analysis of a previously conducted RCT comparing CBM‐APP with a sham control training among patients with PTSD (for full details, see Woud, Blackwell, et al. 2018; Woud et al. 2021). The study recruited a total of 80 inpatients from the Department of Psychosomatic Medicine and Psychotherapy at LWL‐University Clinic, Ruhr‐Universität Bochum, Germany (for demographic characteristics of both groups, see Table 1). The sample size was determined via a priori power analysis to ensure 80% power to detect a moderate‐to‐large between‐group effect (*d* = 0.70, *α* = 0.05). Given the sample size per network (*n* = 37–40), a post hoc power analysis suggests that the present study may detect moderate‐to‐large bivariate associations (*r* ≥ 0.40) at 80% power (*α* = 0.05, two‐tailed). This should, however, not be interpreted as a formal power analysis for Gaussian graphical models, as such analyses depend on unknown network characteristics (e.g., sparsity and true edge distribution), but rather as an approximation to contextualize the detectability of edge weights. All participants received the clinic's standard multimodal PTSD treatment programme and began the study intervention approximately 2 weeks after admission. Eligible participants were adults aged 18–60 years and fluent in German with a primary diagnosis of PTSD (ICD‐10 F43.1; DSM‐5), assessed using the Clinician‐Administered PTSD Scale (CAPS‐5; Cwik et al. 2015; Weathers et al. 2018). Exclusion criteria included suicidality, substance dependence, psychosis and intellectual impairment (Woud, Blackwell, et al. 2018). The CBM‐APP intervention was a computerized cognitive training with 8 training sessions over 2 weeks designed to modify dysfunctional trauma‐related appraisals into more adaptive interpretations. Participants completed positive word fragments resolving ambiguous trauma‐related sentences derived from the PTCI (Foa et al. 1999), covering self‐, world‐ and self‐blame appraisals. Each session consisted of 66 trauma‐related and 15 neutral sentences, lasting approximately 20 min. For our current analyses, data from those participants (*N* = 77) who completed all the relevant measures at both pre‐ and post‐training were used (for the present sample's demographic data, see Table 1). Of the three participants who were excluded, two had not completed the IAT at either pre‐ or post‐training, and one was discharged from the clinic prior to the post‐training assessment and thus provided no post‐training data (see Woud et al. 2021). The original trial was ethically approved by the Faculty of Psychology and Faculty of Medicine at Ruhr‐Universität Bochum. All participants provided informed consent.

**TABLE 1:** Demographics for both groups.

		CBM (*n* = 37)	Control (*n* = 40)
		*n (%) /M* (SD)	*n (%) /M* (SD)
Gender			
	Female	34 (91.9%)	34 (85.0%)
	Male	3 (8.1%)	6 (15.0%)
Age		42.95 (12.23)	38.73 (12.43)
Marital status			
	Single	15 (40.5%)	15 (37.5%)
	Married/civil partnership	14 (37.8%)	15 (37.5%)
	Not living with partner	1 (2.70%)	4 (10.0%)
	Divorced/revoked civil partnership	7 (18.9%)	6 (15.0%)
Marital status			
	Single	15 (40.5%)	15 (37.5%)
Children *n* (%)		17 (45.9%)	19 (47.5%)
Migration background *n* (%)		6 (16.2%)	4 (10.0%)
Native German speaker *n* (%)		33 (89.2%)	37 (92.5%)

### 1.3 Materials

#### 1.3.1 PTSD Symptoms

PTSD symptom severity was measured using the German version of the PTSD Checklist for DSM‐5 (PCL‐5; Krüger‐Gottschalk et al. 2017; Weathers et al. 2013), a 20‐item self‐report questionnaire assessing symptoms across the four DSM‐5 clusters: re‐experiencing, avoidance, negative alterations in cognition and mood and hyperarousal. Participants rated how strongly they experienced each symptom over the past week on a 5‐point Likert scale (0 = *not at all* to 4 = *extremely*). Internal consistencies as reported by Woud et al. (2021) were acceptable‐to‐good with Cronbach's *α* = 0.76 at pretraining and *α* = 0.85 at posttraining. In the current secondary analysis, subscale scores were used to examine changes in PTSD symptoms and their associations with dysfunctional appraisals over time, specifically: Re‐experiencing (Criterion B), avoidance (Criterion C), alterations in cognition and mood (Criterion D) and hyperarousal (Criterion E). We focused on symptom clusters rather than the 20 individual PCL‐5 symptoms to reduce model complexity and ensure a more favourable ratio between sample size and the number of estimated parameters. Given the relatively small sample size, estimating a network with all individual symptoms in addition to the cognitive measures would likely have resulted in unstable edge‐weight and centrality estimates.

#### 1.3.2 Dysfunctional Appraisals

Dysfunctional appraisals were assessed using a scenario completion task administered before and after the intervention (Woud et al. 2019). Participants completed 10 trauma‐related and 4 neutral ambiguous scenarios by writing the first spontaneous ending that came to mind. Responses were coded by two independent raters, blind to condition, for the presence of an appraisal and whether it was dysfunctional (1) or non‐dysfunctional (0). Disagreements were resolved by discussion, yielding a consensus score representing the proportion of dysfunctional appraisals relative to all codable responses. Interrater reliability for dysfunctionality ratings was excellent (96.8% agreement; *κ* = 0.94, 95% CI 0.92–0.96).

#### 1.3.3 Implicit Associations

Implicit automatically activated self‐associations were measured using an Implicit Association Test (IAT; Blackwell et al. 2023; Greenwald et al. 1998; Lindgren et al. 2013; Woud, Blackwell, et al. 2018) designed to assess the strength of associations between the self and trauma‐related attributes. The task required participants to categorize words presented sequentially on a screen into four categories: ‘me’, ‘not me’ (target categories) and ‘traumatized’, ‘healthy’ (attribute categories). In the critical blocks, one target and one attribute category shared a response key, resulting in two key conditions: (1) ‘me’ + ‘traumatized’ vs. ‘not me’ + ‘healthy’, and (2) ‘me’ + ‘healthy’ vs. ‘not me’ + ‘traumatized’. Reaction times served as indicators of associative strength; faster responses when ‘me’ and ‘traumatized’ shared a key reflected stronger implicit identification with a traumatized self. The IAT was administered at baseline and posttraining. The IAT was scored using the D600 method (Greenwald et al. 2003). Specifically, the difference in the mean reaction times for the critical blocks was divided by their pooled standard deviation. Each error trial was replaced by the mean reaction time for the correct trials of that block plus a penalty of 600 ms (see Woud et al. 2021). Split‐half reliability for the IAT, calculated using 5000 randomly‐generated split halves, was 0.80, 95% CIs = [0.68,0.87], at pre‐training, and 0.74 [0.59,0.83] at post‐training (see Woud et al. 2021).

### 1.4 Analysis Plan

Analyses were conducted using R version 4.5.2 (R Core Team 2025). We computed four separate cross‐sectional networks, two for each group, as well as pre‐ and post‐training networks: (1) pre‐training control network, (2) pre‐training CBM network, (3) post‐training control network and (4) post‐training CBM network. Despite randomization, there may be baseline differences in covariance structure among variables. Therefore, we estimated separate networks for each group at baseline. For each network, we estimated a Gaussian Graphical Model (GGM) using the estimateNetwork function from the bootnet package with the *cor_auto* function from qgraph (Epskamp et al. 2018) because the dataset contained mixed measurement levels (ordinal, proportion, and continuous variables). The *cor_auto* function selects pairwise association estimates appropriate to each variable combination based on their distributional and scaling properties (e.g., polychoric correlations for ordinal scores and Pearson correlations for continuous combinations, Epskamp et al. 2018). Note that PCL‐5 scores were treated as ordinal variable (as determined by the cor_auto function). In a GGM, nodes represent symptoms, and edges reflect the partial correlations between pairs of nodes, controlling for all other nodes in the network (Borsboom and Cramer 2013). Green edges represent positive associations and red edges negative associations. Networks were visualized using the qgraph package (Epskamp et al. 2018). We applied the least absolute shrinkage and selection operator (LASSO) regularization with the tuning parameter gamma set to its default value of 0.5 (Foygel and Drton 2010). Differences in overall network connectivity were examined using the Network Comparison Test (NCT; Epskamp et al. 2018). Specifically, we examined differences in global strength (sum of absolute edge weights). For each symptom, we computed expected influence (EI) centrality, which quantifies a node's overall connectedness (i.e., the sum of its edge weights) and thus its relative importance within the network (Robinaugh et al. 2016). To assess the accuracy of edge weights, 95% confidence intervals (CIs) were obtained via nonparametric bootstrapping with 1000 iterations. We further evaluated the stability of centrality estimates using correlation‐stability (CS) analysis. This method correlates centrality indices from the full sample with those derived from subsets of the data (case‐drop bootstrapping, range 0–1; Epskamp et al. 2018). The CS coefficient indicates the proportion of cases that can be removed while maintaining a correlation of at least 0.7 between the original and subset‐based statistics. Values above 0.5 are considered acceptable (Epskamp et al. 2018). Finally, we conducted edge‐weight difference tests and centrality difference tests to determine whether specific symptom connections or node centralities differed significantly from one another (ibid). The edge‐weight test identifies whether certain symptom pairs are more strongly connected than others, whereas the centrality difference test evaluates whether some symptoms are more central (i.e., influential) within the network.

## 2 Results

### 2.1 Descriptive Statistics

Table 2 presents the descriptive statistics of all constructs, separately for the (1) pre‐training control network, (2) pre‐training CBM network, (3) post‐training control network and the (4) post‐training CBM network. There were no pre‐training differences between groups concerning descriptive statistics. Re‐experiencing (Criterion B), alterations in cognition and mood (Criterion D) and dysfunctional appraisals assessed via scenario task significantly improved from pre‐ to post‐training in the CBM group and differed significantly from the control group at post‐training. In the control group, hyperarousal (Criterion E) slightly decreased from pre‐ to post‐training.

**TABLE 2:** Descriptive statistics of all constructs for each of the four networks.

	1. Pre‐control		2. Pre‐CBM		3. Post‐Control		4. Post‐CBM		Comparison			
	*M*	SD	*M*	SD	*M*	SD	*M*	SD	*1* vs. *2*	*1* vs. *3*	*2* vs. *4*	*3* vs. *4*
1. Criterion B	14.63	3.05	14.10	3.84	14.95	3.82	12.62	4.77	ns	ns	*	*
2. Criterion C	5.93	1.60	5.82	1.60	5.40	2.38	4.38	2.35	ns	ns	ns	ns
3. Criterion D	19.47	4.58	19.26	3.71	18.52	4.98	14.93	5.64	ns	ns	**	**
4. Criterion E	15.80	3.91	15.07	3.91	14.40	4.22	12.31	5.30	ns	*	ns	ns
5. IAT	0.16	0.40	0.24	0.42	0.04	0.39	0.02	0.25	ns	ns	ns	ns
6. Scenario task	0.78	0.14	0.80	0.16	0.72	0.24	0.37	0.32	ns	ns	***	***

*Note:* Re‐experiencing (Criterion B), Avoidance (Criterion C), Alterations in Cognition and Mood (Criterion D); Hyperarousal (Criterion E), IAT = implicit association test; Dysfunctional appraisals assessed via scenario task (Scenario task). *N* = 40 for control and *N* = 37 for the CBM group.

*** *p* < 0.001.

** *p* < 0.01.

* *p* < 0.05, ns = non‐significant.

### 2.2 Network Stability

Network stability tests pointed to only moderate stability of the networks. Accuracy plots showed rather wide CIs, indicating only weak‐to‐moderate edge weight stability (Figures S1–S4). Case‐drop bootstrapping results indicated weak‐to‐moderate stability of EI centrality measures (Figures S5–S8). Finally, the correlation stability coefficient was 0.28 for the pre‐training control network, 0.22 for the pre‐training CBM network, 0.35 for the post‐training CBM network and 0.43 for the post‐training CBM network, indicating low stability of the estimates, as all coefficients were below the recommended cut‐off of 0.50. Accordingly, the following results should be interpreted with caution.

### 2.3 Network Comparisons

According to the NCT, there were no significant differences in overall connectivity (differences in global strength) among the four networks, all *p*s > 0.59. The rank‐order of edge weights (i.e., the edge lists) showed weak‐to‐strong correlations across networks. Specifically, the pre‐training control and pre‐training CBM networks were weakly correlated, *r* = 0.23, *p* = 0.213. The pre‐training and post‐training control networks showed a moderate correlation, *r* = 0.33, *p* = 0.077. The pre‐training and post‐training CBM networks were strongly correlated, *r* = 0.76, *p* < 0.001. Finally, the post‐training control and post‐training CBM networks were moderately correlated, *r* = 0.35, *p* = 0.055.

Similarly, the rank‐order of EI centrality across networks also showed weak‐to‐strong correlations. The pre‐training control and pre‐training CBM networks were weakly correlated, *r* = 0.30, *p* = 0.569. The pre‐training and post‐training control networks were moderately correlated, *r* = 0.45, *p* = 0.367. The pre‐training and post‐training CBM networks were strongly correlated, *r* = 0.75, *p* = 0.089. Finally, the post‐training control and post‐training CBM networks were strongly correlated, *r* = 0.93, *p* = 0.001.

### 2.4 Edge Weights

**FIGURE 1:**
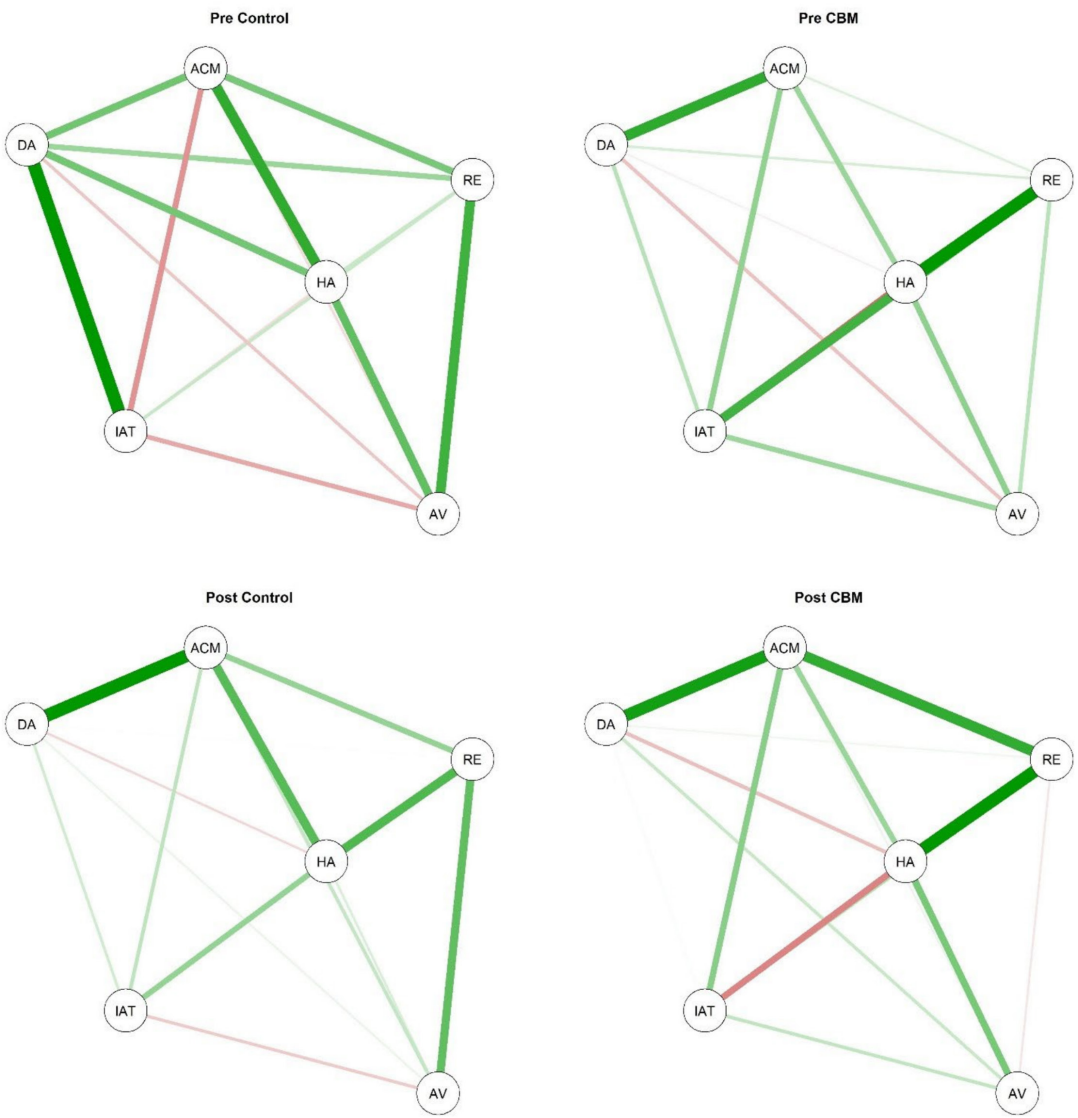
Cross‐sectional networks for the pre‐treatment control group, the pre‐treatment CBM‐group, the post‐treatment control group and the post‐treatment CBM group. *Note:* RE = Re‐experiencing (Criterion B), AV = Avoidance (Criterion C), ACM = Alterations in Cognition and Mood (Criterion D); HA = Hyperarousal (Criterion E), IAT = implicit association test; DA = Dysfunctional appraisals assessed via scenario task. We used an average layout across all four cross‐sectional networks. This means that nodes of all three networks were placed at the same position so that the networks can be more easily compared visually. Green edges represent positive associations and red edges negative associations.

Figure 1 presents the network models. Figures S9–S12 display the edge weight difference plots, and Data S2–S5 provide the complete edge lists.

In the pre‐training control network, the strongest edges were observed between *Dysfunctional appraisals (scenario task)* and *IAT* (*r* = 0.43), *Alterations in Cognition and Mood* and *Hyperarousal (Criterion D)* (*r* = 0.35) and between *Re‐experiencing (Criterion B)* and *Avoidance (Criterion C)* (*r* = 0.32).

In the pre‐training *CBM* network, the strongest associations were between *Re‐experiencing (Criterion B)* and *Hyperarousal (Criterion E)* (*r* = 0.48), *Alterations in Cognition and Mood (Criterion D)* and *Dysfunctional appraisals (scenario task)* (*r* = 0.39) and between *Re‐experiencing* and *IAT* (*r* = 0.36).

In the post‐training control network, the strongest edges were found between *Alterations in Cognition and Mood* and *Dysfunctional appraisals (scenario task)* (*r* = 0.52), *Re‐experiencing (Criterion B)* and *Hyperarousal (Criterion E)* (*r* = 0.36) and between *Alterations in Cognition and Mood (Criterion D)* and *Hyperarousal (Criterion E)* (*r* = 0.34).

Finally, in the post‐training CBM network, the strongest associations were between *Re‐experiencing (Criterion B)* and *Hyperarousal (Criterion E)* (*r* = 0.50), *Alterations in Cognition and Mood (Criterion D)* and *Dysfunctional appraisals (scenario task)* (*r* = 0.46) and between *Re‐experiencing (Criterion B)* and *Alterations in Cognition and Mood (Criterion D)* (*r* = 0.40).

### 2.5 EI Centrality

**FIGURE 2:**
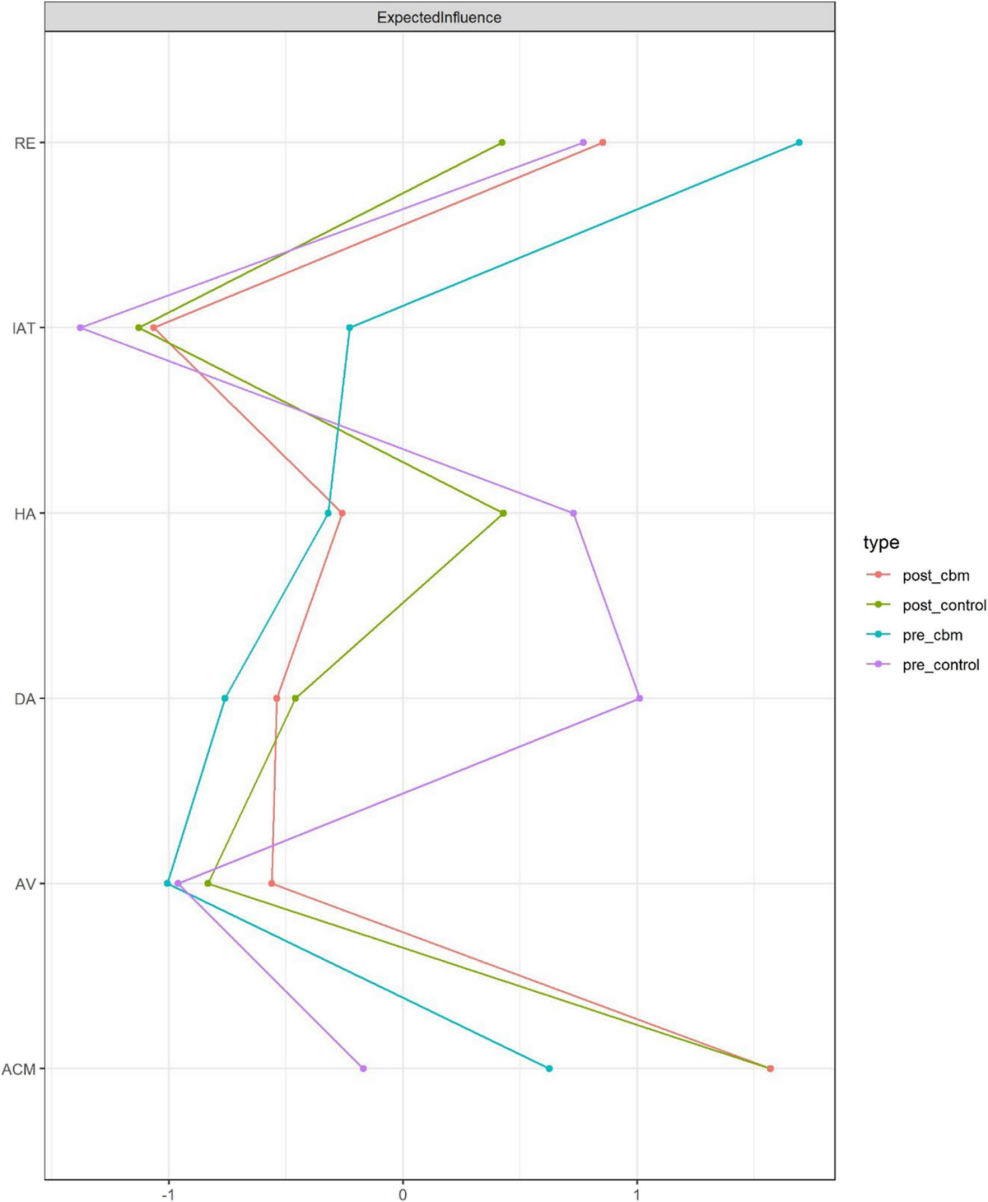
Expected influence centrality plot for (1) the pre‐training control group, (2) the pre‐training CBM‐group, (3) the post‐training control group and (4) the post‐treatment CBM group. *Note:* Numbers on the x‐axis present z‐values. RE = Re‐experiencing (Criterion B), AV = Avoidance (Criterion C), ACM = Alterations in Cognition and Mood (Criterion D); HA = Hyperarousal (Criterion E), IAT = implicit association test; DA = Dysfunctional appraisals assessed via scenario task.

The EI centrality of each construct is presented in Figure 2. Differences among EI centrality within networks are depicted in the EI difference plots in Figures S13–S16. There were no significant differences between EI centrality across networks.

In the pre‐training control network, *Dysfunctional appraisals (scenario task)* showed the highest centrality, followed by *Re‐experiencing (Criterion B)* and *Hyperarousal (Criterion E)*. In the pre‐training CBM network, *Re‐experiencing (Criterion B)* had the highest strength centrality, followed by *Alterations in Cognition and Mood (Criterion D)*. In the post‐training control network, *Alterations in Cognition and Mood (Criterion D)* exhibited the highest strength centrality, followed by *Hyperarousal* and *Re‐experiencing*. Finally, in the *post‐training CBM network*, *Alterations in Cognition and Mood* again showed the highest EI, followed by *Re‐experiencing*.

Importantly, the EI of the *IAT* in the pre‐training CBM network decreased descriptively at follow‐up, while the centrality of *Dysfunctional appraisals (scenario task)* decreased from the pre‐training control network to the post‐training assessment.

## 3 Discussion

This secondary network analysis examined the interplay among PTSD symptom clusters and multiple cognitive processing levels before and after CBM‐APP, a training that has been shown to reduce dysfunctional appraisals and PTSD symptoms (Woud et al. 2021). Our study incorporated measures of reflective appraisal and automatic trauma‐related associations. Findings from the present study offer preliminary insights into putative mechanisms by which cognitive training may influence PTSD‐related processes in interventions. Networks showed only weak‐to‐moderate stability. No significant differences in overall connectivity were observed across networks. Across networks, PTSD symptom subscales measured by the PCL5, *Re‐experiencing (Criterion B)* and *Alterations in Cognition & Mood (Criterion D)* consistently showed high centrality, with *Alterations in Cognition & Mood* being the most central symptom in both post‐training networks, whereas self‐trauma associations measured by the *IAT* and *Dysfunctional appraisals* (scenario task; i.e., measured by an open‐ended scenario completion task) decreased in centrality from pre‐ to post‐training in the CBM and control networks, respectively.

Overall, the networks showed low stability for centrality indices and edge‐weight estimates, which is likely attributable to the small sample size in the context of the present secondary analyses. Therefore, the present findings should be regarded as preliminary and interpreted with utmost caution. Replication in larger samples is needed before we can draw solid conclusions about the centrality of cognitive processes or the impact of the intervention on network connectivity (Epskamp et al. 2018). No meaningful differences in overall network connectivity were observed between pre‐ and post‐training networks or between CBM‐APP and control groups. This may indicate that the intervention did not substantially alter the overall structure of cognitive‐emotional networks. However, given the small sample and low stability of centrality indices, the study may have been underpowered to detect subtle changes.

Across networks, *Re‐experiencing* (Criterion B) and *Alterations in Cognition and Mood* (Criterion D) consistently showed high centrality, highlighting the relevant link between both criteria. This aligns with cognitive models of PTSD, which propose that persistent symptoms arise from dysfunctional appraisals and trauma‐related memory representations (Ehlers and Clark 2000; McNally and Woud 2019). The high centrality of *Re‐experiencing* indicates its potential to activate other symptom clusters through intrusive memories, which is in line with previous PTSD network analysis (yet, previous network analyses have produced heterogeneous results, for a review, see Birkeland et al. 2020). However, we cannot determine the directionality of these associations. These symptoms may have emerged as highly central symptoms because they present precursor symptoms that instigate and sustain other symptoms, or recipient symptoms that emerge as a downstream consequence of broader network activation (Borsboom et al. 2021; McNally 2021). For instance, *Re‐experiencing* could precipitate avoidance or hyperarousal symptoms, but they may also be amplified by existing negative beliefs or emotional numbing. Importantly, this distinction is crucial for interventions: If these symptoms represent precursor symptoms, treatments that directly target dysfunctional appraisals or facilitate trauma memory integration (e.g., cognitive restructuring, trauma exposure or directly targeting intrusive memories, e.g., Kanstrup et al. 2024) may disrupt symptom escalation throughout the network (Snyder et al. 2015). Conversely, if they are recipient symptoms of other processes, interventions may need to prioritize antecedent mechanisms, such as emotion regulation or avoidance reduction, to achieve optimal outcomes (ibid). Future longitudinal or temporal network analyses are needed to clarify potential causal pathways and inform intervention targets accordingly (e.g., Schlechter et al. 2022).

At baseline, networks showed random differences between groups. *Dysfunctional appraisals* measured by the scenario task were more central in the control group, whereas the *IAT* had higher centrality in the pre‐CBM‐APP network. After training, these patterns shifted. In the CBM‐APP group, *IAT* centrality decreased, becoming comparable to the pre‐ and post‐control network, suggesting a targeted reduction in the influence of automatic dysfunctional self‐associations. In the control group, scenario‐based dysfunctional appraisals decreased in centrality over time. However, these findings were descriptive and non‐significant, due to the low power of our sample. The decrease in *IAT* centrality relative to other nodes following CBM‐APP (note that it was not among the most central symptoms in the pre‐training network) may indicate that the training may have selectively weakened the correlations between automatic self‐associations and other PTSD symptoms. CBM‐APP engages reflective appraisal processes through repeated exposure to trauma‐related scenarios that systematically resolve in adaptive ways (Woud et al. 2012, 2013). The post‐training reduction in IAT centrality suggests that automatic dysfunctional associations exert less influence on symptom activation. More adaptive reflective appraisals due to CBM‐APP may override previously dominant automatic associations, potentially decreasing their influence on network‐wide symptom activation. Importantly, mean IAT scores did not significantly change from pre‐ to post‐training, indicating that the implicit association itself may not have changed substantially. Instead, it appears that its relationship with other symptoms and cognitive processes became weaker. Because CBM‐APP primarily targets reflective appraisal processes through repeated reinterpretation of ambiguous trauma‐related scenarios, the reduction in IAT centrality may not reflect a direct change in implicit associations. Rather, more adaptive appraisals may reduce the extent to which automatic negative self‐associations are linked to other PTSD symptoms. This interpretation differs from more bottom‐up CBM paradigms, which directly target automatic processing tendencies. Thus, CBM‐APP may primarily alter the functional role of implicit associations within the symptom network rather than the associations themselves. Future longitudinal research is needed to determine whether CBM‐APP can change implicit self‐trauma associations directly or mainly reduce their influence on other PTSD symptoms.

In both post‐training networks, *Alterations in Cognition and Mood* (Criterion D) emerged as the most central node and even slightly increased in centrality compared to pre‐training. High centrality indicates that activation of this symptom can instigate symptom escalation across the network (McNally 2021). However, this increase does not necessarily imply a maladaptive shift in network centrality in the CBM‐APP group. Mean levels of this symptom were significantly lower in the CBM‐group both from pre‐ to post‐training and relative to the control group. As CBM‐APP applied in clinical contexts aims to reduce dysfunctional appraisals, the fact that Criterion D was a central symptom suggests that individuals are less likely to activate this node. Functionally, this reflects a network increasingly regulated by adaptive appraisal pathways, as the node is structurally central but less frequently engaged. In this regard, dysfunctional appraisals could act as a gateway mechanism that initiates and maintains broader network activation. Interventions like CBM‐APP that recalibrate these appraisals may therefore interrupt the cascading activation of symptoms, or lead to lower levels of activation which is in turn associated with lower overall network activation.

### 3.1 Clinical Implications

Given the small sample size and only modest network stability, any clinical implications should be interpreted as tentative and primarily informative for future research rather than direct clinical guidance. Theoretically, highly central nodes may serve as leverage points for treatment (Kleim et al. 2013). Interventions targeting cognitive appraisals could have cascading effects across PTSD symptoms. Modifying appraisal processes, as in CBM‐APP, may thus reduce activation across interconnected symptom clusters. Assessing individual symptom network structure before intervention may be important, as baseline differences in centrality (such as higher *IAT* centrality in the pre‐CBM network) could moderate treatment response. Personalized, network‐informed interventions could enhance treatment efficacy by focusing on the most influential nodes. Longitudinal and experimental network studies are yet needed to clarify causal pathways, testing whether altering central nodes like Criterion D consistently reduces network activity or whether certain nodes serve as more effective targets for specific symptom profiles (Henry et al. 2022; McNally 2021).

### 3.2 Strengths and limitations

Our study examined network‐level changes following CBM‐APP in PTSD. By including dysfunctional appraisals, automatically activated self‐associations and core symptom clusters, our analyses capture potentially clinically relevant mechanisms and their interconnections. The design allows for observation of pre‐ to post‐training shifts, offering preliminary evidence for how CBM may alter symptom network structures.

However, there are important limitations to our study. First, while statistical power was sufficient for the initial RCT (Woud et al. 2021), statistical power for the present secondary analysis was weak‐to‐moderate as reflected by weak‐to‐moderate stability of centrality indices and edge‐weight estimates in the secondary network analysis. This, in turn, increases the likelihood that observed network patterns may be influenced by random variability rather than reflecting true underlying relationships. Second, baseline differences in the networks (note that there were no differences in general descriptive statistics) between groups suggest some degree of random variability, which may affect the interpretation of pre‐ to post‐training changes. Third, the networks are correlational. As such, causal inferences about the mechanisms of CBM‐APP cannot be drawn (Borsboom et al. 2021). While changes in centrality may reflect intervention effects, they may also be influenced by unmeasured confounding variables or spontaneous symptom fluctuations. Relatedly, analyses were exploratory and not pre‐registered. Fourth, the study focused on a limited set of cognitive and symptom constructs. Other potentially relevant factors such as comorbid psychiatric conditions, trauma type or severity, or behavioural and physiological indicators were not included. Excluding these variables may reduce the comprehensiveness of the network model and limit the generalizability of findings. Fifth, measurement limitations should be considered. The scenario task and IAT provide useful indices of reflective and automatic appraisals (Blackwell et al. 2023; Woud et al. 2019), respectively, but each captures only a portion of the cognitive processes relevant to PTSD. Consequently, measurement error or task‐specific effects may influence node centrality estimates. In addition, the evidence regarding the IAT's reliability and construct validity is mixed (Schimmack 2021), although we note that split‐half reliability in the current study was good. Accordingly, its use as an indicator of implicit cognition should be interpreted with caution given its psychometric limitations. Finally, the generalizability of these findings is limited. Participants were drawn from a relatively homogeneous patient sample, and it is unclear whether similar network patterns or intervention effects would be observed in more diverse clinical populations. In this regard, we lacked more detailed indicators of socioeconomic background variables. Future research should replicate these findings in larger, more diverse samples, using multi‐method assessments to strengthen confidence in the robustness and clinical relevance of network‐based conclusions.

### 3.3 Conclusion

The present study provides preliminary evidence that CBM‐APP may influence the relative centrality of cognitive and symptom constructs in PTSD networks. Post‐training, *Alterations in Cognition and Mood* emerged as the most central node, suggesting that targeting key cognitive processes could have cascading effects on symptom activation. Changes in *IAT* centrality indicate potential cross‐modality effects. Given the limited statistical power and modest network stability, replications and extensions are needed to confirm whether cognitive training can meaningfully reshape PTSD symptom networks.

## Funding

This research was funded by a postdoctoral scholarship of the Daimler and Benz Foundation (32‐12/4) awarded to the last author M.L.W. The Daimler and Benz Foundation had no role in study design, data collection and analysis, decision to publish, or preparation of the manuscript.

## Conflicts of Interest

The authors declare no conflicts interest.

## Supporting information


**Data S1:** Supporting Information.
**Figure S1:** Edge weight accuracy in the pre‐training control network.
**Figure S2:** Edge weight accuracy in the pre‐training CBM network.
**Figure S3:** Edge weight accuracy in the post‐training control network.
**Figure S4:** Edge weight accuracy in the post‐training CBM network.
**Figure S5:** Centrality stability in the pre‐training control network.
**Figure S6:** Centrality stability in the pre‐training CBM network.
**Figure S7:** Centrality stability in the post‐training control network.
**Figure S8:** Centrality stability in the post‐training CBM network.
**Figure S9:** Edge weight difference tests in the pre‐training control network.
**Figure S10:** Edge weight difference tests in the pre‐training CBM network.
**Figure S11:** Edge weight difference tests in the post‐training control network.
**Figure S12:** Edge weight difference tests in the post‐training CBM network.
**Figure S13:** Expected influence difference tests in the pre‐training control network.
**Figure S14:** Expected influence difference tests in the pre‐training CBM network.
**Figure S15:** Expected influence difference tests in the post‐training control network.
**Figure S16:** Expected influence difference tests in the post‐training CBM network.

**Data S2:** Supplemental Material 2: Edge list pre‐training control network.

**Data S3:** Supplemental Material 3: Edge list pre‐training CBM network.

**Data S4:** Supplemental Material 4: Edge list post‐training control network.

**Data S5:** Supplemental Material 5: Edge list post‐training CBM network.

## Data Availability

The data and R code used in this study are openly available: https://osf.io/jvstf/.
