# Supplementary material for: Does Cognitive Bias Modification for Appraisals Change Symptom‐Cognition Relations in PTSD? Preliminary Evidence from Network Analysis in a Randomized Controlled Trial
Source: Clin Psychol Psychother. 2026 Jul 18;33(4):e70308. doi: 10.1002/cpp.70308 (PMC13379729; doi:10.1002/cpp.70308)
Supplement: Supplementary file 1 — Data S1: Supporting Information. Figure S1: Edge weight accuracy in the pre‐training control network. Figure S2: Edge weight accuracy in the pre‐training CBM network. Figure S3: Edge weight accuracy in the post‐training control network. Figure S4: Edge weight accuracy in the post‐training CBM network. Figure S5: Centrality stability in the pre‐training control network. Figure S6: Centrality stability in the pre‐training CBM network. Figure S7: Centrality stability in the post‐training control network. Figure S8: Centrality stability in the post‐training CBM network. Figure S9: Edge weight difference tests in the pre‐training control network. Figure S10: Edge weight difference tests in the pre‐training CBM network. Figure S11: Edge weight difference tests in the post‐training control network. Figure S12: Edge weight difference tests in the post‐training CBM network. Figure S13: Expected influence difference tests in the pre‐training control network. Figure S14: Expected influence difference tests in the pre‐training CBM network. Figure S15: Expected influence difference tests in the post‐training control network. Figure S16: Expected influence difference tests in the post‐training CBM network. [file CPP-33-e70308-s003.docx]

**
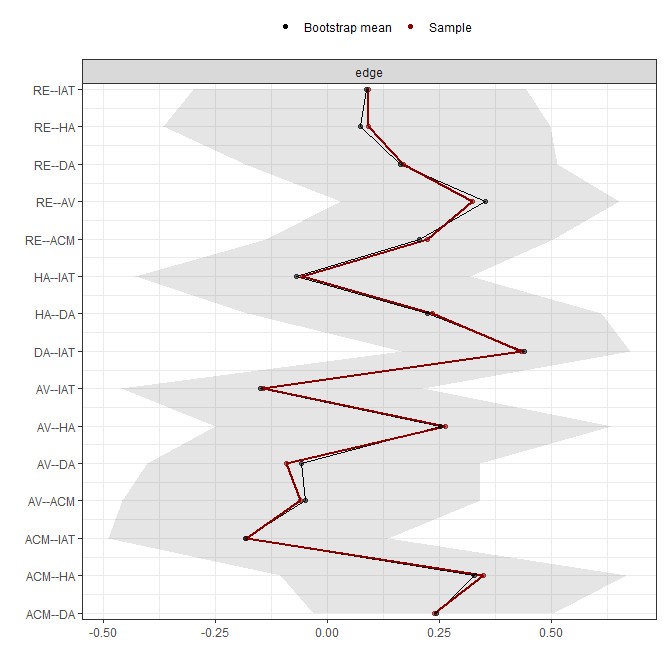
**

**Figure s1.** Interrelation accuracy plots with 1000 bootstrap iterations for the pre-training control network. Plots show the sample interrelations (i.e., edge weights) with the red dots, the means of the bootstrapped interrelations (i.e., edge weights) with black dots, and the bootstrap confidence intervals.

RE = Re-experiencing (Criterion B), AV = Avoidance (Criterion C), ACM = Alterations in Cognition and Mood (Criterion D); HA = Hyperarousal (Criterion E), IAT = implicit association test; DA = Dysfunctional appraisals assessed via scenario task

**
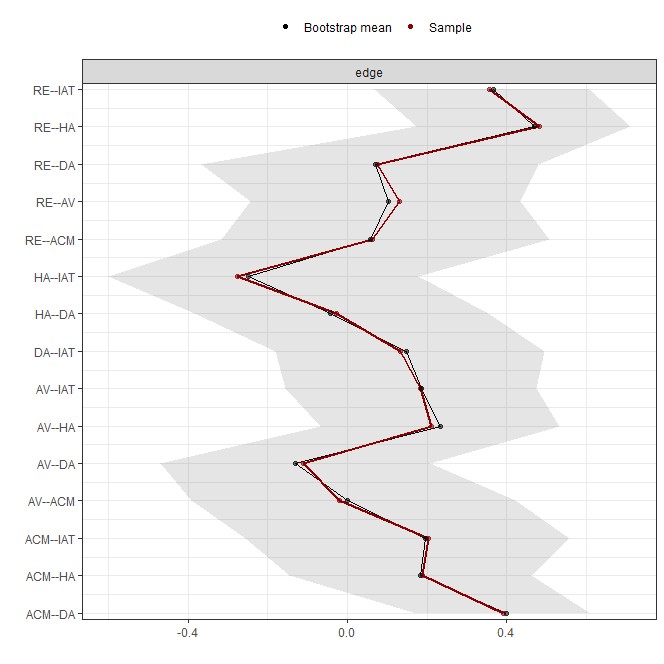
**

**Figure s2.** Interrelation accuracy plots with 1000 bootstrap iterations for the pre-training CBM network. Plots show the sample interrelations (i.e., edge weights) with the red dots, the means of the bootstrapped interrelations (i.e., edge weights) with black dots, and the bootstrap confidence intervals.

RE = Re-experiencing (Criterion B), AV = Avoidance (Criterion C), ACM = Alterations in Cognition and Mood (Criterion D); HA = Hyperarousal (Criterion E), IAT = implicit association test; DA = Dysfunctional appraisals assessed via scenario task

**
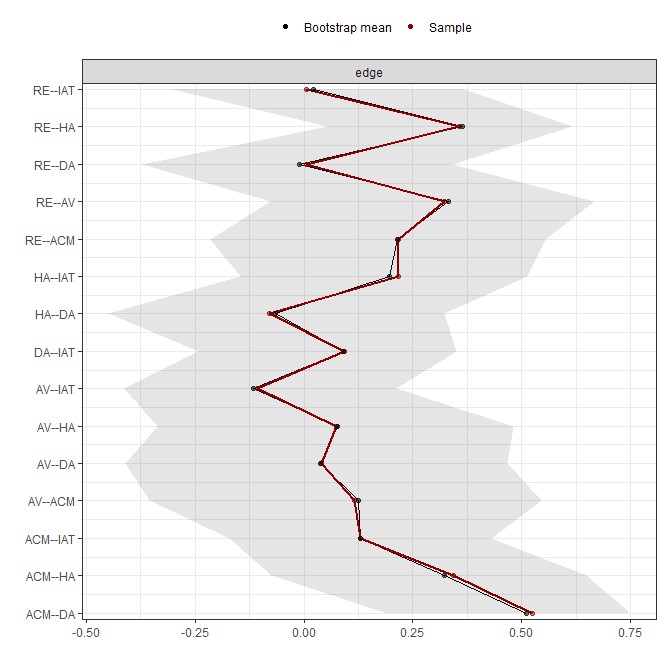
**

**Figure s3.** Interrelation accuracy plots with 1000 bootstrap iterations for the post-training control network. Plots show the sample interrelations (i.e., edge weights) with the red dots, the means of the bootstrapped interrelations (i.e., edge weights) with black dots, and the bootstrap confidence intervals.

RE = Re-experiencing (Criterion B), AV = Avoidance (Criterion C), ACM = Alterations in Cognition and Mood (Criterion D); HA = Hyperarousal (Criterion E), IAT = implicit association test; DA = Dysfunctional appraisals assessed via scenario task

**
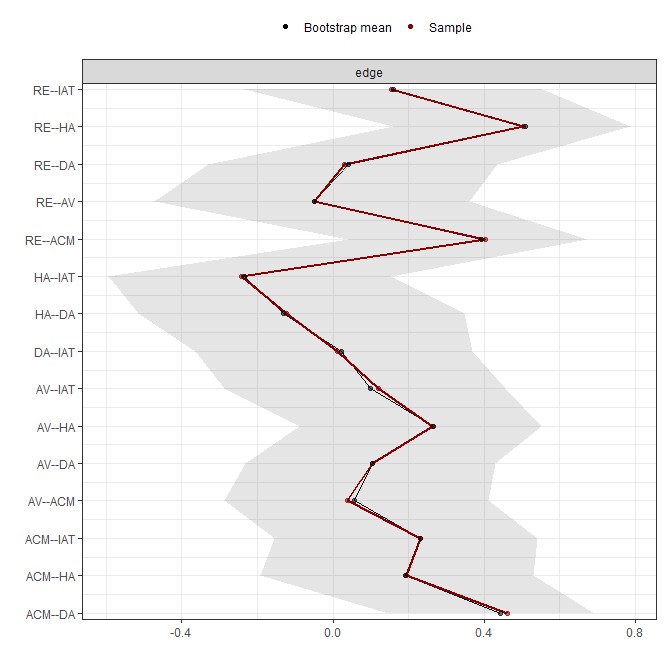
**

**Figure s4.** Interrelation accuracy plots with 1000 bootstrap iterations for the post-training CBM network. Plots show the sample interrelations (i.e., edge weights) with the red dots, the means of the bootstrapped interrelations (i.e., edge weights) with black dots, and the bootstrap confidence intervals.

RE = Re-experiencing (Criterion B), AV = Avoidance (Criterion C), ACM = Alterations in Cognition and Mood (Criterion D); HA = Hyperarousal (Criterion E), IAT = implicit association test; DA = Dysfunctional appraisals assessed via scenario task

**
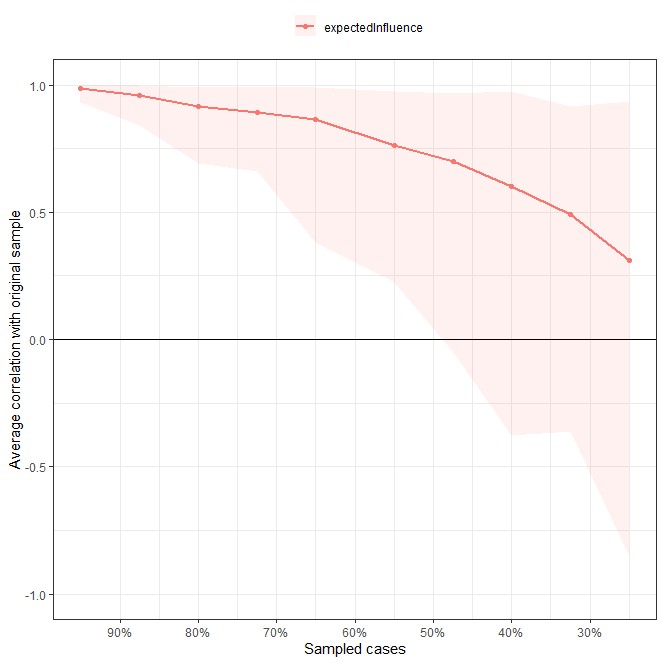
**

**Figure s5.** Stability of the Expected influence centrality measures for the pre-training control network

RE = Re-experiencing (Criterion B), AV = Avoidance (Criterion C), ACM = Alterations in Cognition and Mood (Criterion D); HA = Hyperarousal (Criterion E), IAT = implicit association test; DA = Dysfunctional appraisals assessed via scenario task

**
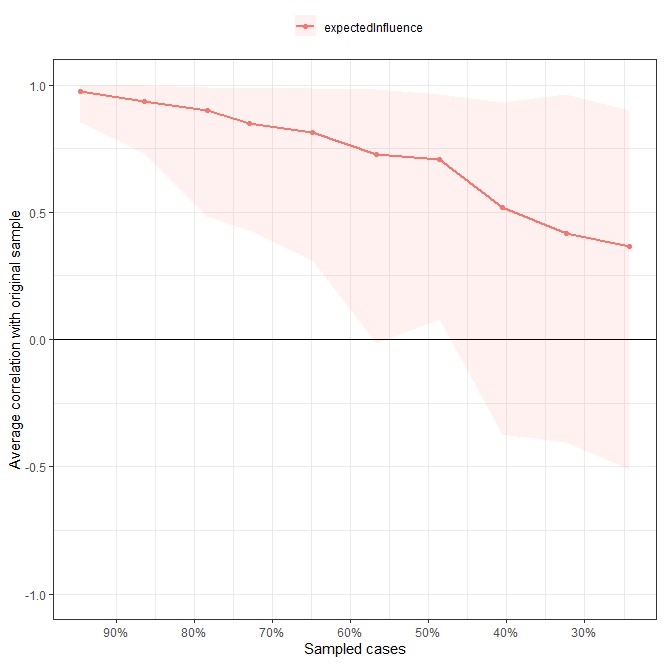
**

**Figure s6.** Stability of the Expected influence centrality measures for the pre-training CBM network

RE = Re-experiencing (Criterion B), AV = Avoidance (Criterion C), ACM = Alterations in Cognition and Mood (Criterion D); HA = Hyperarousal (Criterion E), IAT = implicit association test; DA = Dysfunctional appraisals assessed via scenario task

**
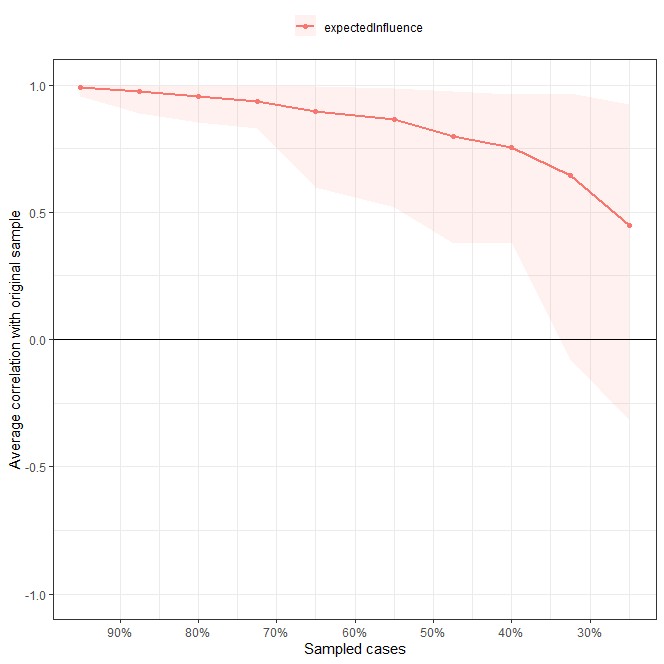
**

**Figure s7.** Stability of the Expected influence centrality measures for the post-training control network

RE = Re-experiencing (Criterion B), AV = Avoidance (Criterion C), ACM = Alterations in Cognition and Mood (Criterion D); HA = Hyperarousal (Criterion E), IAT = implicit association test; DA = Dysfunctional appraisals assessed via scenario task

**
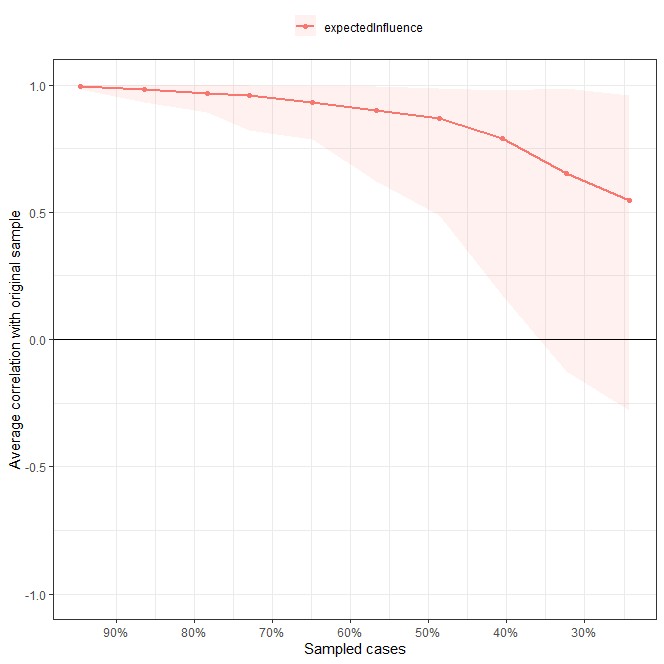
**

**Figure s8.** Stability of the Expected influence centrality measures for the post-training CBM network

RE = Re-experiencing (Criterion B), AV = Avoidance (Criterion C), ACM = Alterations in Cognition and Mood (Criterion D); HA = Hyperarousal (Criterion E), IAT = implicit association test; DA = Dysfunctional appraisals assessed via scenario task


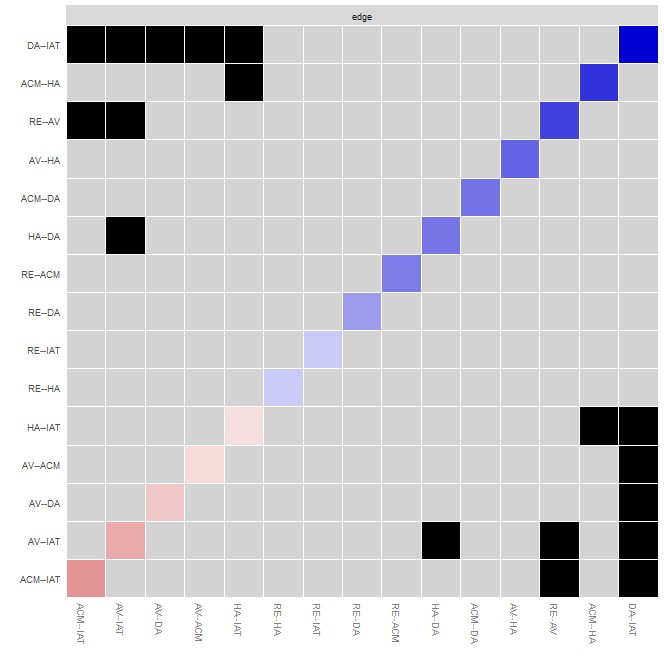


**Figure s9.** Edge weight difference tests for the pre-training control network. Black boxes indicate edges that significantly differ from each other (*p* < .05). Gray boxes indicate no differences. Diagonal indicates the magnitude of the associations: blue (upper right-hand corner) indicates a positive correlation, white indicates a correlation of zero, while pink (lower left-hand corner) indicates a negative correlation. Correlations are ordered by the magnitude of their association, with darker shades indicating stronger associations compared with lighter shades (the coefficients can be found in the edge list in Supplemental Material 2).

RE = Re-experiencing (Criterion B), AV = Avoidance (Criterion C), ACM = Alterations in Cognition and Mood (Criterion D); HA = Hyperarousal (Criterion E), IAT = implicit association test; DA = Dysfunctional appraisals assessed via scenario task


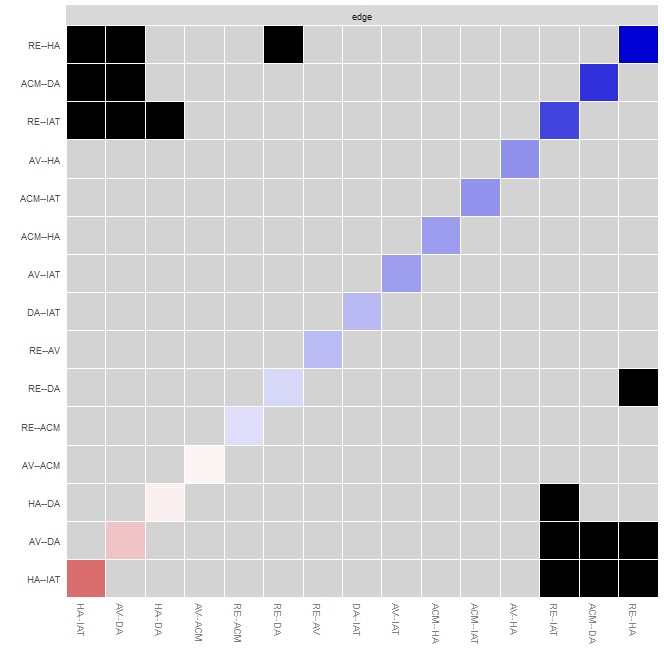


**Figure s10.** Edge weight difference tests for the pre-training CBM network. Black boxes indicate edges that significantly differ from each other (*p* < .05). Gray boxes indicate no differences. Diagonal indicates the magnitude of the associations: blue (upper right-hand corner) indicates a positive correlation, white indicates a correlation of zero, while pink (lower left-hand corner) indicates a negative correlation. Correlations are ordered by the magnitude of their association, with darker shades indicating stronger associations compared with lighter shades (the coefficients can be found in the edge list in Supplemental Material 3).

RE = Re-experiencing (Criterion B), AV = Avoidance (Criterion C), ACM = Alterations in Cognition and Mood (Criterion D); HA = Hyperarousal (Criterion E), IAT = implicit association test; DA = Dysfunctional appraisals assessed via scenario task

**
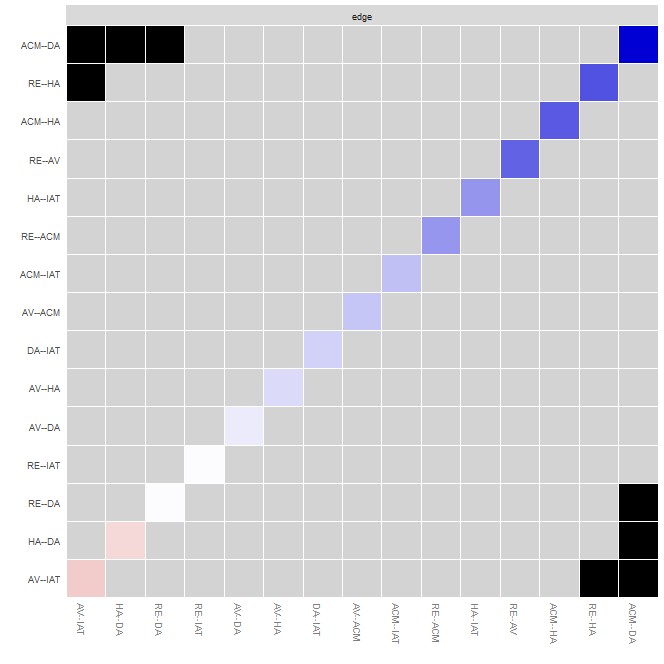
**

**Figure s11.** Edge weight difference tests for the post-training control network. Black boxes indicate edges that significantly differ from each other (*p* < .05). Gray boxes indicate no differences. Diagonal indicates the magnitude of the associations: blue (upper right-hand corner) indicates a positive correlation, white indicates a correlation of zero, while pink (lower left-hand corner) indicates a negative correlation. Correlations are ordered by the magnitude of their association, with darker shades indicating stronger associations compared with lighter shades (the coefficients can be found in the edge list in Supplemental Material 4).

RE = Re-experiencing (Criterion B), AV = Avoidance (Criterion C), ACM = Alterations in Cognition and Mood (Criterion D); HA = Hyperarousal (Criterion E), IAT = implicit association test; DA = Dysfunctional appraisals assessed via scenario task

**
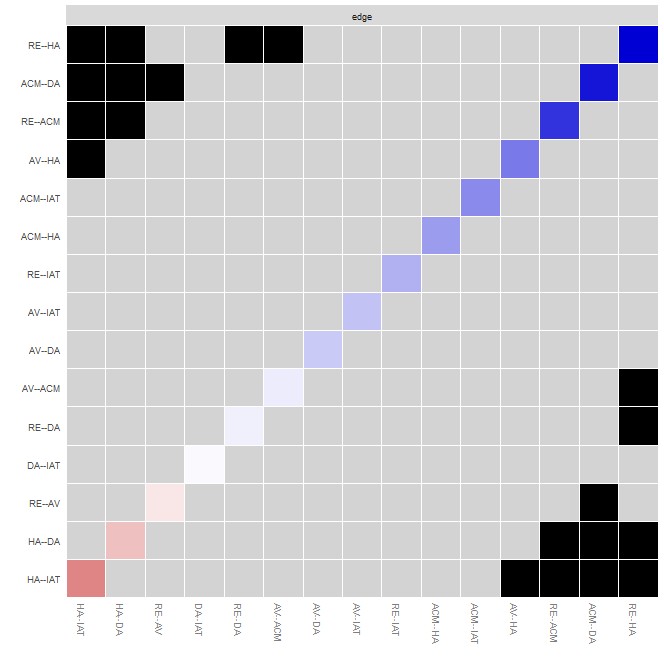
**

**Figure s12.** Edge weight difference tests for the post-training CBM network. Black boxes indicate edges that significantly differ from each other (*p* < .05). Gray boxes indicate no differences. Diagonal indicates the magnitude of the associations: blue (upper right-hand corner) indicates a positive correlation, white indicates a correlation of zero, while pink (lower left-hand corner) indicates a negative correlation. Correlations are ordered by the magnitude of their association, with darker shades indicating stronger associations compared with lighter shades (the coefficients can be found in the edge list in Supplemental Material 5).

RE = Re-experiencing (Criterion B), AV = Avoidance (Criterion C), ACM = Alterations in Cognition and Mood (Criterion D); HA = Hyperarousal (Criterion E), IAT = implicit association test; DA = Dysfunctional appraisals assessed via scenario task

**
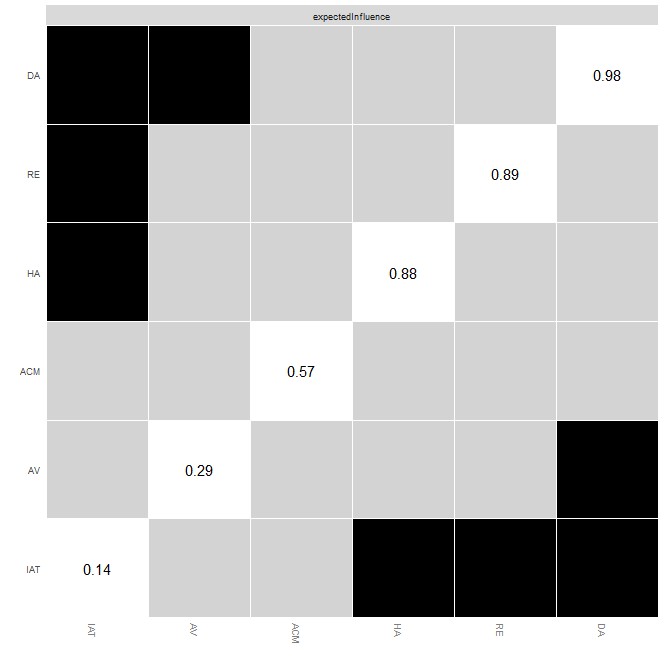
**

**Figure s13.** Expected influence difference tests for the pre-training control network. Black boxes indicate nodes that significantly differ from each other (*p* < .05). Gray boxes indicate no differences.

RE = Re-experiencing (Criterion B), AV = Avoidance (Criterion C), ACM = Alterations in Cognition and Mood (Criterion D); HA = Hyperarousal (Criterion E), IAT = implicit association test; DA = Dysfunctional appraisals assessed via scenario task

**
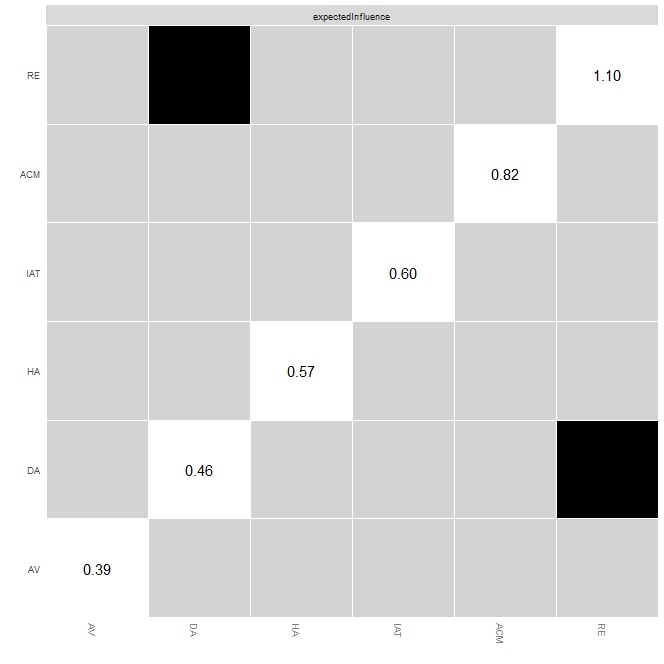
**

**Figure s14.** Expected influence difference tests for the pre-training CBM network. Black boxes indicate nodes that significantly differ from each other (*p* < .05). Gray boxes indicate no differences.

RE = Re-experiencing (Criterion B), AV = Avoidance (Criterion C), ACM = Alterations in Cognition and Mood (Criterion D); HA = Hyperarousal (Criterion E), IAT = implicit association test; DA = Dysfunctional appraisals assessed via scenario task

**
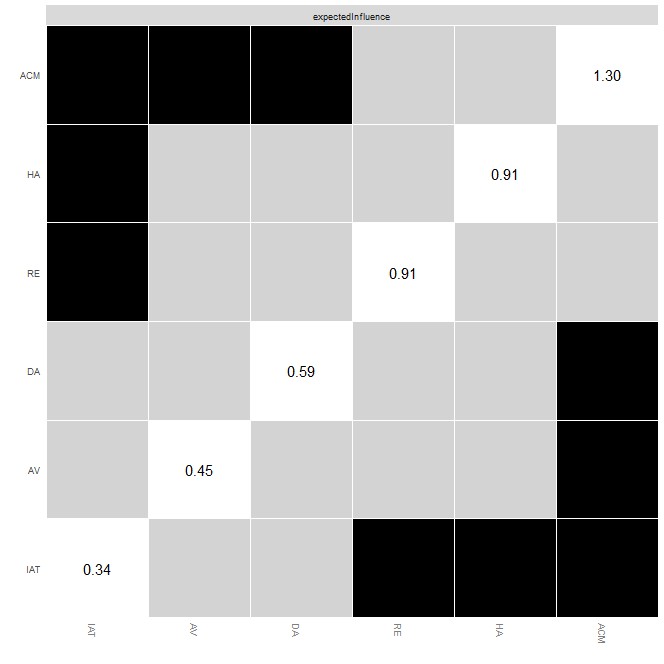
**

**Figure s15.** Expected influence difference tests for the post-training control network. Black boxes indicate nodes that significantly differ from each other (*p* < .05). Gray boxes indicate no differences.

RE = Re-experiencing (Criterion B), AV = Avoidance (Criterion C), ACM = Alterations in Cognition and Mood (Criterion D); HA = Hyperarousal (Criterion E), IAT = implicit association test; DA = Dysfunctional appraisals assessed via scenario task


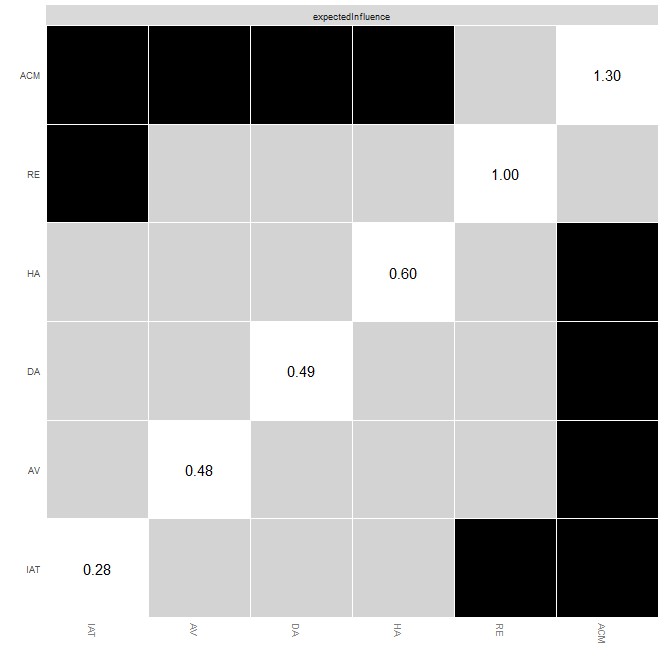


**Figure s16.** Expected influence difference tests for the post-training CBM network. Black boxes indicate nodes that significantly differ from each other (*p* < .05). Gray boxes indicate no differences.

RE = Re-experiencing (Criterion B), AV = Avoidance (Criterion C), ACM = Alterations in Cognition and Mood (Criterion D); HA = Hyperarousal (Criterion E), IAT = implicit association test; DA = Dysfunctional appraisals assessed via scenario task
