# Supplementary material for: Does Cognitive Bias Modification for Appraisals Change Symptom‐Cognition Relations in PTSD? Preliminary Evidence from Network Analysis in a Randomized Controlled Trial
Source: Clin Psychol Psychother. 2026 Jul 18;33(4):e70308. doi: 10.1002/cpp.70308 (PMC13379729; doi:10.1002/cpp.70308)
Supplement: Supplementary file 2 — Data S2: Supplemental Material 2: Edge list pre‐training control network. [file CPP-33-e70308-s002.pdf]

	name	type	node1	node2	value	id
15	sample	edge	DA	IAT	0,43159337	DA--IAT
6	sample	edge	ACM	HA	0,34712365	ACM--HA
1	sample	edge	RE	AV	0,32207441	RE--AV
5	sample	edge	AV	HA	0,26387397	AV--HA
9	sample	edge	ACM	DA	0,23750733	ACM--DA
10	sample	edge	HA	DA	0,233359	HA--DA
2	sample	edge	RE	ACM	0,22259895	RE--ACM
7	sample	edge	RE	DA	0,16822654	RE--DA
11	sample	edge	RE	IAT	0,09101991	RE--IAT
4	sample	edge	RE	HA	0,09052421	RE--HA
14	sample	edge	HA	IAT	-0,05521214	HA--IAT
3	sample	edge	AV	ACM	-0,06103298	AV--ACM
8	sample	edge	AV	DA	-0,09236948	AV--DA
12	sample	edge	AV	IAT	-0,1435148	AV--IAT
13	sample	edge	ACM	IAT	-0,18063398	ACM--IAT

nNode	nPerson	rank_avg	rank_min	rank_max	graph
6	40	15	15	15	1
6	40	14	14	14	1
6	40	13	13	13	1
6	40	12	12	12	1
6	40	11	11	11	1
6	40	10	10	10	1
6	40	9	9	9	1
6	40	8	8	8	1
6	40	7	7	7	1
6	40	6	6	6	1
6	40	5	5	5	1
6	40	4	4	4	1
6	40	3	3	3	1
6	40	2	2	2	1
6	40	1	1	1	1
