# Supplementary material for: Does Cognitive Bias Modification for Appraisals Change Symptom‐Cognition Relations in PTSD? Preliminary Evidence from Network Analysis in a Randomized Controlled Trial
Source: Clin Psychol Psychother. 2026 Jul 18;33(4):e70308. doi: 10.1002/cpp.70308 (PMC13379729; doi:10.1002/cpp.70308)
Supplement: Supplementary file 3 — Data S3: Supplemental Material 3: Edge list pre‐training CBM network. [file CPP-33-e70308-s004.pdf]

	name	type	node1	node2	value	id
4	sample	edge	RE	HA	0,48194509	RE--HA
9	sample	edge	ACM	DA	0,3904105	ACM--DA
11	sample	edge	RE	IAT	0,35592394	RE--IAT
5	sample	edge	AV	HA	0,21058335	AV--HA
13	sample	edge	ACM	IAT	0,20421497	ACM--IAT
6	sample	edge	ACM	HA	0,18680833	ACM--HA
12	sample	edge	AV	IAT	0,1838026	AV--IAT
15	sample	edge	DA	IAT	0,13163807	DA--IAT
1	sample	edge	RE	AV	0,12979387	RE--AV
7	sample	edge	RE	DA	0,07593604	RE--DA
2	sample	edge	RE	ACM	0,06155009	RE--ACM
3	sample	edge	AV	ACM	-0,01920935	AV--ACM
10	sample	edge	HA	DA	-0,02805217	HA--DA
8	sample	edge	AV	DA	-0,11137562	AV--DA
14	sample	edge	HA	IAT	-0,27678537	HA--IAT

nNode	nPerson	rank_avg	rank_min	rank_max	graph
6	37	15	15	15	1
6	37	14	14	14	1
6	37	13	13	13	1
6	37	12	12	12	1
6	37	11	11	11	1
6	37	10	10	10	1
6	37	9	9	9	1
6	37	8	8	8	1
6	37	7	7	7	1
6	37	6	6	6	1
6	37	5	5	5	1
6	37	4	4	4	1
6	37	3	3	3	1
6	37	2	2	2	1
6	37	1	1	1	1
