# Supplementary material for: Does Cognitive Bias Modification for Appraisals Change Symptom‐Cognition Relations in PTSD? Preliminary Evidence from Network Analysis in a Randomized Controlled Trial
Source: Clin Psychol Psychother. 2026 Jul 18;33(4):e70308. doi: 10.1002/cpp.70308 (PMC13379729; doi:10.1002/cpp.70308)
Supplement: Supplementary file 4 — Data S4: Supplemental Material 4: Edge list post‐training control network. [file CPP-33-e70308-s005.pdf]

	name	type	node1	node2	value	id
9	sample	edge	ACM	DA	0,52530742	ACM--DA
4	sample	edge	RE	HA	0,35759691	RE--HA
6	sample	edge	ACM	HA	0,34229184	ACM--HA
1	sample	edge	RE	AV	0,32248737	RE--AV
14	sample	edge	HA	IAT	0,21745435	HA--IAT
2	sample	edge	RE	ACM	0,21683387	RE--ACM
13	sample	edge	ACM	IAT	0,12945924	ACM--IAT
3	sample	edge	AV	ACM	0,11654437	AV--ACM
15	sample	edge	DA	IAT	0,09316569	DA--IAT
5	sample	edge	AV	HA	0,07509661	AV--HA
8	sample	edge	AV	DA	0,04092428	AV--DA
11	sample	edge	RE	IAT	0,00669877	RE--IAT
7	sample	edge	RE	DA	0,00669478	RE--DA
10	sample	edge	HA	DA	-0,08023692	HA--DA
12	sample	edge	AV	IAT	-0,10662569	AV--IAT

nNode	nPerson	rank_avg	rank_min	rank_max	graph
6	40	15	15	15	1
6	40	14	14	14	1
6	40	13	13	13	1
6	40	12	12	12	1
6	40	11	11	11	1
6	40	10	10	10	1
6	40	9	9	9	1
6	40	8	8	8	1
6	40	7	7	7	1
6	40	6	6	6	1
6	40	5	5	5	1
6	40	4	4	4	1
6	40	3	3	3	1
6	40	2	2	2	1
6	40	1	1	1	1
