# Supplementary material for: Does Cognitive Bias Modification for Appraisals Change Symptom‐Cognition Relations in PTSD? Preliminary Evidence from Network Analysis in a Randomized Controlled Trial
Source: Clin Psychol Psychother. 2026 Jul 18;33(4):e70308. doi: 10.1002/cpp.70308 (PMC13379729; doi:10.1002/cpp.70308)
Supplement: Supplementary file 5 — Data S5: Supplemental Material 5: Edge list post‐training CBM network. [file CPP-33-e70308-s001.pdf]

| | name | type | node1 | node2 | value | id |
|---|---|---|---|---|---|---|
| 4 | sample | edge | RE | HA | 0,50436357 | RE--HA |
| 9 | sample | edge | ACM | DA | 0,46249762 | ACM--DA |
| 2 | sample | edge | RE | ACM | 0,4044253 | RE--ACM |
| 5 | sample | edge | AV | HA | 0,26439439 | AV--HA |
| 13 | sample | edge | ACM | IAT | 0,23109096 | ACM--IAT |
| 6 | sample | edge | ACM | HA | 0,19517961 | ACM--HA |
| 11 | sample | edge | RE | IAT | 0,15420957 | RE--IAT |
| 12 | sample | edge | AV | IAT | 0,12015187 | AV--IAT |
| 8 | sample | edge | AV | DA | 0,10561738 | AV--DA |
| 3 | sample | edge | AV | ACM | 0,03730403 | AV--ACM |
| 7 | sample | edge | RE | DA | 0,030194 | RE--DA |
| 15 | sample | edge | DA | IAT | 0,01198354 | DA--IAT |
| 1 | sample | edge | RE | AV | -0,0493926 | RE--AV |
| 10 | sample | edge | HA | DA | -0,12432326 | HA--DA |
| 14 | sample | edge | HA | IAT | -0,24122596 | HA--IAT |

| nNode | nPerson | rank_avg | rank_min | rank_max | graph |
|---|---|---|---|---|---|
| 6 | 37 | 15 | 15 | 15 | 1 |
| 6 | 37 | 14 | 14 | 14 | 1 |
| 6 | 37 | 13 | 13 | 13 | 1 |
| 6 | 37 | 12 | 12 | 12 | 1 |
| 6 | 37 | 11 | 11 | 11 | 1 |
| 6 | 37 | 10 | 10 | 10 | 1 |
| 6 | 37 | 9 | 9 | 9 | 1 |
| 6 | 37 | 8 | 8 | 8 | 1 |
| 6 | 37 | 7 | 7 | 7 | 1 |
| 6 | 37 | 6 | 6 | 6 | 1 |
| 6 | 37 | 5 | 5 | 5 | 1 |
| 6 | 37 | 4 | 4 | 4 | 1 |
| 6 | 37 | 3 | 3 | 3 | 1 |
| 6 | 37 | 2 | 2 | 2 | 1 |
| 6 | 37 | 1 | 1 | 1 | 1 |
